# De Novo Mining and Validating Novel Microsatellite Markers to Assess Genetic Diversity in *Maruca vitrata* (F.), a Legume Pod Borer

**DOI:** 10.3390/genes14071433

**Published:** 2023-07-12

**Authors:** Rashmi Manohar Mahalle, Tejas C. Bosamia, Snehel Chakravarty, Kartikeya Srivastava, Radhe S. Meena, Ulhas Sopanrao Kadam, Chandra P. Srivastava

**Affiliations:** 1 Department of Entomology and Agricultural Zoology, Institute of Agricultural Sciences, Banaras Hindu University, Varanasi 221005, India; 2 Department of Applied Biology, College of Agriculture and Life Sciences, Chungnam National University, Daejeon 34134, Republic of Korea; 3 Plant Omics Division, Central Salt and Marine Chemicals Research Institute, Bhavnagar 364002, India; 4 Department of Genetics and Plant Breeding, Institute of Agricultural Sciences, Banaras Hindu University, Varanasi 221005, India; 5 Plant Molecular Biology and Biotechnology Research Center (PMBBRC), Division of Life Science and Applied Life Science (BK21 Four), Gyeongsang National University, Jinju 52828, Republic of Korea

**Keywords:** pigeonpea, legume pod borer, expressed sequence tag, microsatellites, diversity

## Abstract

*Maruca vitrata* (Fabricius) is an invasive insect pest capable of causing enormous economic losses to a broad spectrum of leguminous crops. Microsatellites are valuable molecular markers for population genetic studies; however, an inadequate number of *M. vitrata* microsatellite loci are available to carry out population association studies. Thus, we utilized this insect’s public domain databases for mining expressed sequence tags (EST)-derived microsatellite markers. In total, 234 microsatellite markers were identified from 10053 unigenes. We discovered that trinucleotide repeats were the most predominant microsatellite motifs (61.53%), followed by dinucleotide repeats (23.50%) and tetranucleotide repeats (14.95%). Based on the analysis, twenty-five markers were selected for validation in *M. vitrata* populations collected from various regions of India. The number of alleles (*Na*), observed heterozygosity (*Ho*), and expected heterozygosity (*He*) ranged from 2 to 5; 0.00 to 0.80; and 0.10 to 0.69, respectively. The polymorphic loci showed polymorphism information content (*PIC*), ranging from 0.09 to 0.72. Based on the genetic distance matrix, the unrooted neighbor-joining dendrogram differentiated the selected populations into two discrete groups. The SSR markers developed and validated in this study will be helpful in population-level investigations of *M. vitrata* to understand the gene flow, demography, dispersal patterns, biotype differentiation, and host dynamics.

## 1. Introduction

The spotted pod borer, *M. vitrata*, first described by Fabricius in 1787, belongs to Lepidoptera (Crambidae). It is a serious global concern of leguminous crops in sub-Saharan Africa, tropical Asia, Australia, America, and the Pacific regions [1]. This pest species devastates at least 73 host plant species, including cowpea, soybean, lablab bean, adzuki bean, and black gram, accounting for approximately 72% yield losses [2]. It has been recorded as a significant pest in nine legume crops, whereas in Asia, the pigeonpea (*Cajanus cajan*), is its principal host [3]. The larvae feed on the buds, flowers, developing pods, and pigeonpea leaves. The annual economic loss in pigeonpea caused specifically by *M*. *vitrata* globally has been estimated to be at least USD 30 million [4]. It is also reported to cause yield losses of 20 to 80% in infested pigeonpea fields and may lead to total crop failure without proper insect population management [3]. Legume farmers rely primarily on chemical insecticides as the primary control method to tackle *M. vitrata* damage [5]. However, the extensive utilization of insecticides has increased resistance in this pest species against them, thereby leading to population outbreaks and rendering its management more difficult [6].

Population genetic studies can provide insights into the existing genetic variability and genetic structure of targeted pest populations [7], help to comprehend pest invasion history and its capacity to expand in the future [8], and be used for designing and optimizing sustainable pest management strategies [9]. Genetic markers like microsatellites and mitochondrial DNA (mtDNA) markers are extensively employed in population genetic studies to accurately identify species and assess population genetic diversity and genetic differentiation studies. In *M. vitrata*, mitochondrial DNA (*cox1*) was utilized in different studies to track the introduced populations’ relationship and history [4,5,10]. Genetic diversity analysis using *cox1* indicated the existence of three mitochondrial lineages of *Maruca* spp.; the first and second lineages were found in West Africa, Taiwan, and Australia, whereas the third lineage was in Puerto Rico [11]. Furthermore, another study provided evidence supporting three putative *Maruca* species, including one in Latin America, one in Oceania (including Indonesia), and *M. vitrata* in Asia, Africa, and Oceania [10]. This finding suggests distinct lineages within the *Maruca* species, each adapted to specific geographic regions. The study highlights the importance of recognizing these species’ differences and their distributions to enhance our knowledge of *Maruca* diversity worldwide. However, mitochondrial studies provide a single perspective, as mitochondrial DNA has a uniparental inheritance. Furthermore, the assumption of nearly neutral evolution and clock-like evolutionary rate of mtDNA has been questioned, as it fails to account for adaptive processes and deviations from neutrality, making it necessary to consider other genetic markers for a comprehensive understanding of population and species history [12]. Thus, more informative molecular markers such as microsatellites or simple sequence repeats (SSRs) are now preferred for population genetic studies to explore invasive populations’ diversity, migratory patterns, and origin [13].

SSRs are usually 2–7 base pairs in length and have been found to be very useful for population genetics studies owing to their co-dominant and highly polymorphic nature, with relatively excellent genome coverage [14,15,16]. Agunbiade et al. [15] isolated six polymorphic microsatellite loci in *M. vitrata* and efficiently utilized these markers in the population structure analysis of *M. vitrata* from Nigeria, Niger, and Burkina Faso. However, further enrichment is not reported in *M. vitrata* SSRs numbers, and only six microsatellite loci need to be increased for research associated with population genetics. As very little information on the population genetic structure of *M. vitrata*, particularly from the Indian subcontinent, is available, the development of microsatellites was urgent for genetic diversity analyses. The traditional methods of microsatellite development are expensive, time-consuming, and labor-intensive [17]. In silico SSR mining from the public domain is an efficient and cost-effective approach for the identification of microsatellite loci with the availability of next-generation sequencing databases and bioinformatics tools [18,19]. In the present study, the publicly available transcriptome and EST databases of *M. vitrata* were utilized for microsatellite loci mining and validation among geographically isolated populations of *M. vitrata* from India. The polymorphic markers from this study will also help to carry out further advanced studies related to the population genetics of *M. vitrata*.

## 2. Materials and Methods

### 2.1. Ethics Statement

No specific permission was required to sample and collect the *M. vitrata* larvae from the studied locations. The insects used for this study are non-endangered and non-protected species. We also confirm that the selected sampling sites were unprotected and not privately owned.

### 2.2. Insect Sampling and DNA Extraction

Larvae of *M. vitrata* were collected from 20 locations covering India’s four major pigeonpea growing zones during 2018–2019 (Table 1). Around 100 larvae were collected and preserved in 95% ethanol from each collection site. The genomic DNA was extracted separately from four individual late instar larvae of each location. Extraction was carried out from the larval skin using the CTAB method, according to Periasamy et al. [10]. The quality of the extracted DNA samples was checked on 0.8% agarose gel (*w*/*v*) and quantified using NanoDrop ND-1000 (NanoDrop products, Wilmington, DE, USA). The working DNA concentrations (20 ng μL^−1^) were made by pooling the DNA from four individuals from each location. Thus, 80 individuals were used, including 16 individuals from the North East Plain Zone (NEPZ), 20 individuals from the North West Plain Zone (NWPZ), 20 individuals from the Central Zone (CZ), and 24 individuals from the South Zone (SZ).

### 2.3. SSR Mining and Primer Designing

The sequence data available publicly from the NCBI database (http://www.ncbi.nlm.nih.gov/) (accessed on 19 October 2019) was used for SSR mining. The 454 pyrosequencing transcriptome data of *M. vitrata* was also retrieved from the Sequence Read Archive (SRA) of NCBI with SRX032895 (accession number) under BioProject accession PRJNA7970149 [20]. In addition to transcriptome data, *M. vitrata* nucleotide data from NCBI was used to generate assembly (date accessed—19 December 2019). The low-complexity regions, low-quality sequences (<100 bp), tracts of poly-A and poly-T, and the undetermined bases-rich sequence ends were trimmed using Trim-Galore available in the Galaxy tool (https://usegalaxy.org/, accessed on 19 October 2019). Trinity software package with a default parameter on PSC’s Bridges was used to perform the de novo assembly of high-quality reads. To develop novel microsatellites, 10,053 unigenes were generated by assembling the combined datasets of nucleotide sequences and transcriptomes available at NCBI. These unigenes were subjected to the MISA (MIcroSAtellite) Perl script software [21]. The primers were designed based on the following parameters: the maximum number of bases (100 bp) interrupting two compound SSRs and unit size (2–6, 3–5, 4–4, 5–3, and 6–3). The online software BatchPrimer3 v1.0 [22] was utilized to design EST-SSR primer pairs with the following criteria: (1) optimum primer length 20 bp (range: 18–23 bp); (2) optimum Tm 60 °C (range: 57–63 °C); (3) maximum 1.5 °C difference between forward and reverse primer Tm; (4) GC content—optimum 50% (range: 40–60%); and (5) product size—optimum 150 bp (range: 100–300 bp).

### 2.4. Polymerase Chain Reaction (PCR) Amplification

A set of 25 SSR primer pairs were selected randomly from 79 newly developed primers (Table 2 and Table S1) and screened on 20 different geographic populations of *M. vitrata* across India to estimate polymorphism across studied populations. The newly designed primers were synthesized from Eurofins India Pvt. Ltd., Bengaluru, India. The PCR mixture (25 μL) contained two μL template DNA (20 ng), 2.5 μL 10x Taq buffer with MgCl_2_ (GeNei^TM^, Bengaluru, India), 0.8 μL dNTP (2 mM; GeNei^TM^, Bengaluru, India), 2.0 μL forward and reverse primer, each (10 p moles), 0.2 μL Taq polymerase (GeNei^TM^, Bengaluru, India), and 15.3 μL sterile double-distilled water. Thermo cycling consisted of the following steps: initial denaturation at 94 °C for 3 min, followed by 35 cycles of amplification, each cycle with the following schedule: denaturation for 30 s at 94 °C, annealing for 30 s at 60 °C, and extension for 1 min at 72 °C. The final extension was performed at 72 °C for 5 min. Amplified products were examined using 6% non-denaturing polyacrylamide gel stained with ethidium bromide and documented in an automated gel documentation system (Bio-Rad Gel Doc^TM^ XR+, USA). Finally, it was manually scored for the marker polymorphism. A 50 bp DNA ladder (GeNei^TM^, Bengaluru, India) was used to estimate the size range of each SSR amplified in the samples, and this was documented manually. Stutter and background bands were excluded.

### 2.5. Genetic Diversity and Population Structure Assessment

The gene diversity (*H*), major allele frequency (*MAF*), and polymorphism information content (*PIC*) were calculated using the software Power Marker v.3.25 [23]. To estimate genetic diversity indices for polymorphic loci, we calculated the number of alleles (*Na*), the number of effective alleles (*Ne*), unbiased heterozygosity (*uHe*), expected heterozygosity (*He*), observed heterozygosity (*Ho*), fixation index (*F*), Shannon information index (*I*), and Hardy–Weinberg equilibrium with GenAlEx version 6.5 software [24]. Null alleles were estimated using the MICRO-CHECKER [25]. An admixture model was implemented to infer the genetic structure of the populations in STRUCTURE 2.3.4 software [26]. This approach was used to explore the numbers of different clusters (K) and assigns each individual to a cluster based on microsatellite data. The assumption was made that allele frequencies of different populations were correlated. Then, 30 independent runs were performed for each cluster (*K)* value (from 1 to 5) with 250,000 burn-in and 750,000 Markov chain Monte Carlo (MCMC) steps. The STRUCTURE analysis output was then used in the Structure Harvester Web 0.6.94, and the best *K*-value was computed with the Δ*K* method [27]. The online software program CLUMPAK (Cluster Markov Packager Across *K*) was used to summarize each K’s repeat runs and generate the schematic representation of the inferred populations.

Principal coordinates analysis (PCoA) based on the genetic distance was conducted using GenAlEx (version 6.5) via covariance with standardization to detect and plot the similarity genetic distance matrix among individuals [24]. The population genetic variance was further analyzed by AMOVA (Analysis of Molecular Variance) in GenAlEx software (6.5 version) with 999 permutations. A two-part AMOVA analysis was conducted to check genetic divergence (*F_ST_*) as a variation factor among and within the populations. To test whether genetic differentiation among populations followed an isolation-by-distance (IBD) pattern, the Mantel test was performed using the pairwise geographical distance (Ln km) against pairwise linearized genetic distance among individuals in GenAlEx (version 6.5) with 1000 random permutations. The pairwise *F* statistics (*F_ST_*) were estimated among the populations using GenAlEx version 6.5 to measure the probable degree of genetic differentiation. Further, to explore the hierarchical relationships among different *M. vitrata* populations, a neighbor-joining tree was constructed in POPTREE2 software with 1000 bootstraps [28] based on genetic distance. We also performed a ‘Discriminant Analysis of Principal Components (DAPC)’ analysis using Adegenet 4.1.3 implemented in R.

## 3. Results

### 3.1. Identification and Characterization of EST-SSR Motifs

A total of 234 EST-SSRs were detected from 196 unigenes with a frequency of 2.33%. Among these, 24 unigenes possessed more than one EST-SSR, and a total of 13 (5.56%) SSRs were identified as compound microsatellites (Table 3). Among 234 SSRs, trinucleotide repeats were the predominant motif type (144, 61.53%), followed by dinucleotide repeats (55, 23.50%), and tetranucleotide repeats (14.95%). The EST-SSRs with six tandem repeats (93, 39.74%) were the most abundant, followed by five tandem repeats (91, 38.89%). There were also 50 EST-SSRs with over six repeat units (Table 4). Further, 18 motif types were identified among the detected EST-SSRs that comprised 3, 9, and 6 types of dinucleotides, trinucleotides, and tetranucleotides, respectively. The most abundant motif in the dinucleotide repeats was AC/GT (31, 56.36%), followed by AT/AT (20, 36.36%) and CG/CG (4, 7.27%). Among the trinucleotide repeats, the main motif types were AAT/ATT (26.39%), ACG/CGT (26.39%), ATC/ATG (20.14%), and AAG/CTT (12.50%). The most abundant tetranucleotide repeat unit detected was the AAAT/ATTT (65.71%), followed by ACAG/CTGT (11.43%) (Table 5).

### 3.2. Microsatellite Polymorphisms

A total of 79 EST-SSR primer pairs were designed from the mined SSRs of *M. vitrata* transcriptome and details of newly designed primers are given in Table S1. At random, 25 SSR primers were selected to verify their utility in genetic diversity assessment among 20 different geographic populations of *M. vitrata* from the country (Table 2). Of them, 18 microsatellite markers had polymorphic amplification, two markers were monomorphic, and five primer pairs did not produce any visible amplicon (Supplementary Figure S1). The number of alleles (*Na*) and effective alleles (*Ne*) ranged from 2 to 5 and 1.10 to 3.27, respectively, with an average of 2.86 and 1.92. The observed heterozygosity (*Ho*) ranged from 0.00 to 0.80, with an average of 0.34, whereas the expected heterozygosity (*He*) ranged from 0.10 to 0.69, with an average of 0.42. The polymorphism information content values of polymorphic loci ranged between 0.09 and 0.72, averaging 0.45. Shannon information index (*I*) was 0.72, ranging from 0.20 to 1.25, and mean gene diversity was 0.49 ranging from 0.10 to 0.76 (Table 6).

### 3.3. Population Genetic Diversity

The population genetic diversity analysis of *M. vitrata* showed the existence of moderate genetic diversity (Table 7) among populations. The number of effective alleles (*Ne*) ranged from 1.60 to 1.80, with a mean of 1.72. *He* values ranged from 0.31 (NEPZ) to 0.37 (SZ), with an average value of 0.34, whereas *Ho* varied from 0.29 (NEPZ) to 0.35 (SZ), with an average value of 0.32. A positive fixation index (*F_IS_*) was obtained for all zones with a mean of 0.06. *F_IS_* and *F_IT_* values are an indicator to estimate the population nearness, whereas *F_ST_* indicates the genetic differentiation levels among populations [29,30]. The fixation index inbreeding coefficient (*F_IS_*) ranged from 0.02 (‘NWPZ’) to 0.09 (‘NEPZ’). A positive *F_IS_* value reflects an excess of homozygotes (the presence of heterozygotes deficiencies) in these populations [29]. We identified 13 private alleles: four in the ‘NWPZ’ and ‘CZ’, three in the ‘SZ’, and two in ‘NEPZ’ (Table 7).

### 3.4. Population Genetic Differentiation and Variation

The pairwise *F_ST_* estimates between *M. vitrata* populations ranged from 0.029 to 0.065. The highest pairwise *F_ST_* estimate was observed between the ‘NEPZ’ and ‘CZ’ (*F_ST_* = 0.065), whereas the lowest *F_ST_* was recorded between the ‘SZ’ and ‘NWPZ’ populations (Table 8). The pairwise *F_ST_* values were at a relatively higher level for the ‘NEPZ’, with all populations *viz*., ‘NWPZ’ (0.064), ‘CZ’ (0.065), and ‘SZ’ (0.053). However, the lower pairwise *F_ST_* values were observed for ‘SZ’ and ‘CZ’, including 0.049 (between ‘CZ’ and ‘NWPZ’), 0.029 (between ‘SZ’ and ‘NWPZ’), and 0.031 (between ‘CZ’ and ‘SZ’). Following the criterion given by Wright [31], genetic differentiation was considered low for *F_ST_* values less than 0.05, moderate for values between 0.05 and 0.15, high for values between 0.15 and 0.25, and very high for values exceeding 0.25. In the present investigation, gene flow was estimated indirectly from *F_ST_*. It ranged from 3.576 to 8.505, indicating migration among sampling localities. The pairwise population *Nm* value was recorded at 7.932 between ‘SZ’ and ‘CZ’ populations, whereas it was at the highest between ‘NWPZ’ and ‘SZ’ populations (8.505) (Table 8).

### 3.5. Analysis of Molecular Variance (AMOVA)

The result of hierarchical AMOVA revealed that 55% of the total genetic variation existed at the individual level, whereas 40% came from among individuals within populations, and 5% was ascribed to differences among populations. The global *F_ST_* value across all populations was 0.046 (*p* < 0.005), showing the existence of lower population genetic differentiation, whereas the inbreeding coefficient within-population (*F_IS_*) was 0.415 (Table 9), which indicates heterozygosity deficits in populations.

### 3.6. Mantel Test for Isolation by Distance (IBD)

**Figure 1:**
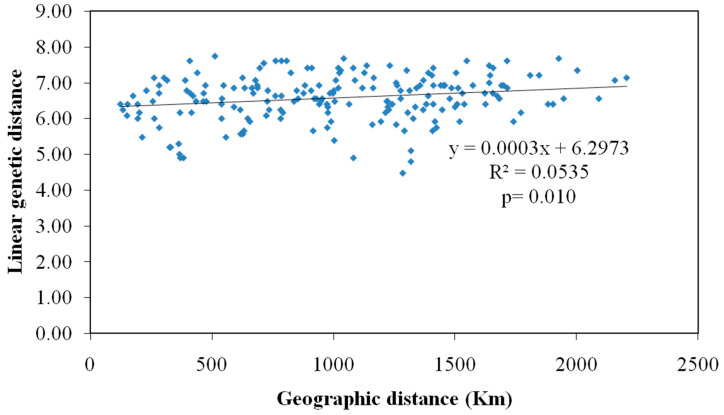
Correlation between genetic and geographical distance among *M. vitrata* populations collected from 20 locations in India.

Mantel’s IBD test was performed to determine the correlation between the genetic distance matrix of studied *M. vitrata* populations with the corresponding geographic distance matrix (Ln km). A non-significant and weak correlation was observed between both variables (R^2^ *=* 0.053, *p =* 0.010) (Figure 1).

### 3.7. Genetic Structure

**Figure 2:**
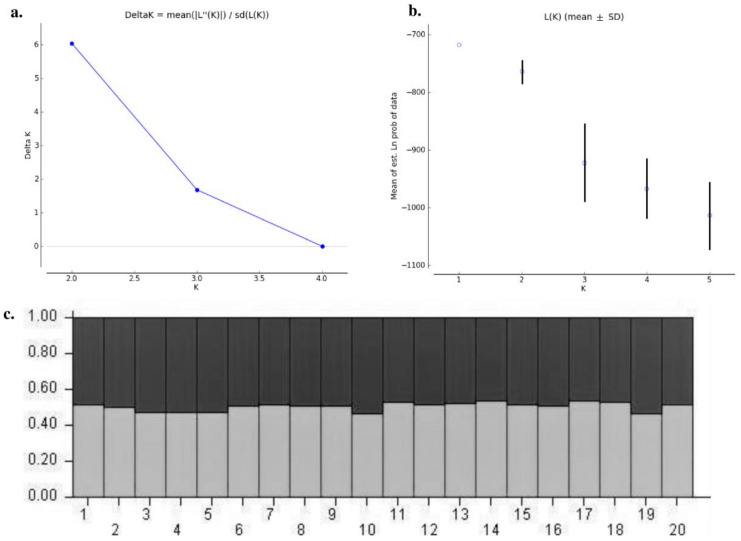
Analysis of the population structure of *M. vitrata* populations collected from 20 locations: (**a**) determination of the optimal value of *K* from Structure Harvester; (**b**) Evanno plot; (**c**) bar plot representations of Bayesian STRUCTURE analysis of *M. vitrata* populations with *K =* 2. The horizontal axis represents the locations codes (1: DMV, 2: AGTL, 3: BSB, 4: KYI, 5: CNB, 6: LDH, 7: HSR, 8: NDLS, 9: PBW, 10: JBP, 11: R, 12: DPLI, 13: LUR, 14: DWZ, 15: BBS, 16: HYB, 17: GNT, 18: RC, 19: DWR, 20: KLBG) displayed as per Table 1.

**Figure 3:**
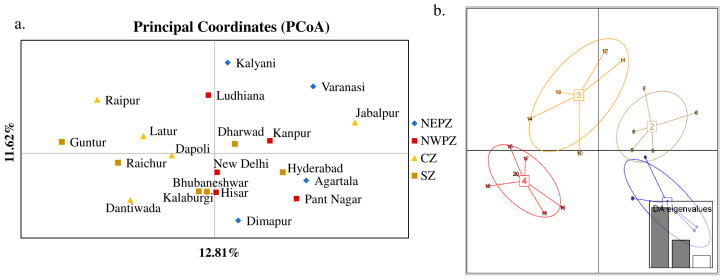
Principal components analysis of *M. vitrata* populations using 29 SSR loci: (**a**) principal coordinates analysis (PCoA); (**b**) Discriminant Analysis of Principal Components (DAPC) in *M. vitrata* populations. The numbers in square brackets indicate insect collection zones. 1—North East Plain Zone, 2—North West Plain Zone, 3—Central Zone, 4—South Zone. The numbers 1–20 are the sampling locations given in Table 1.

**Figure 4:**
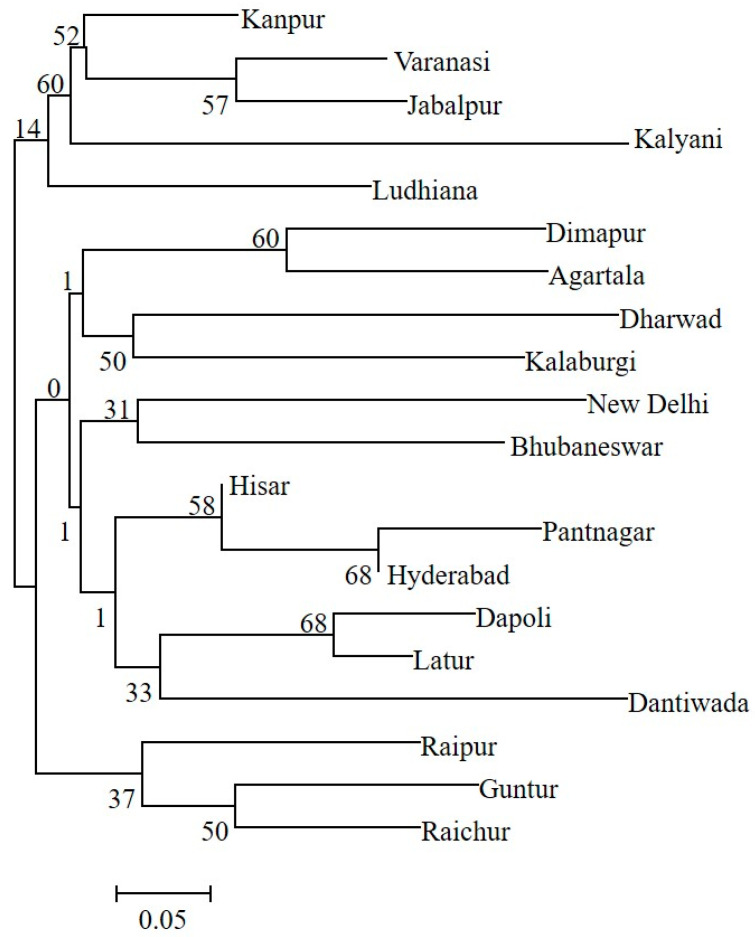
The neighbor-joining tree based on genetic distances shows the clustering of *M. vitrata* populations collected from 20 locations in India. Bootstrap values supporting each branch are indicated on the nodes.

The genetic structure analysis of *M. vitrata* populations inferred using STRUCTURE version 2.3.4 indicated an optimal value of *K =* 2 (Figure 2). All the populations were classified as admixtures at a probability of association of <60%. The genetic structure map, based on the estimated membership probability (Q-matrix), showed that a total of 14 localities belonged to group I (Q-matrix > 0.50), and six localities belonged to group II (Q-matrix < 0.50). Individuals collected from Kanpur, Varanasi, Kalyani, Jabalpur, Ludhiana, and Dharwad formed group II (Figure 2). Principal component analysis (PCoA) showed 12.81% of the total variation for the first principal components, and the second component accounted for 11.62% of the variation. The first three axes explained 35.39% of the cumulative variation (Figure 3a). Six localities, Kanpur, Varanasi, Kalyani, Jabalpur, Ludhiana, and Dharwad, showed close association, whereas individuals from New Delhi, Hyderabad, Agartala, Pantnagar, Dimapur, Hisar, Bhubaneswar, and Kalaburagi were closer to each other. Although in structure and PCA no conclusive group was observed, DAPC analysis also indicated somewhat genetic differentiation among the localities, and the discrepancy between the DAPC and structure results could be due to the differing approaches and assumptions employed by each method. (Figure 3b). The unrooted neighbor-joining dendrogram based on the genetic distance matrix also showed that 20 *M. vitrata* localities were separated into two discrete groups, consistent with the results obtained by PcoA and DAPC analyses (Figure 4). The major cluster was composed of 15 localities and had two sub-clusters. Individuals from Raichur, Guntur, and Raipur were grouped in the same sub-cluster, whereas individuals collected from Kanpur, Varanasi, Kalyani, Jabalpur, Ludhiana, and Dharwad were clustered together.

## 4. Discussion

The molecular markers, particularly microsatellites, have been used extensively for elucidating variations in the population genetic structure of several complex insect pest species of agricultural importance. Here, we performed in silico identification and characterization of SSRs along with validation of newly designed SSRs among *M. vitrata* populations from diverse agroecologies of India. SSR density (one SSR/22 kb) from 234 identified EST-SSR motifs used in the present study is in accordance with previous reports, including the yellow stem borer (*Scirpophaga incertulas*), with a total of 563 EST-SSR motifs having a frequency of one SSR/10.98 kb [32], the onion maggot (*Delia antiqua)* with 332 EST-SSRs in 29,659 unigenes having a frequency of 1 SSR per 14.7 kb [33], and the western corn rootworm (*Diabrotica virgifera*) with 305 SSRs in a database of 6397 EST sequences [14]. The abundance of microsatellite loci shows insect-to-insect variation, such as *Frankliniella occidentalis* (1 SSR/2.9 kb) [19], *Nilaparvata lugens* (1 SSR/13.0 kb) [18], pea aphid (1 SSR/3.6 kb) [34], and *Phenacoccus solenopsis* (1 SSR/2.4 kb) [35]. These differences may be explained due to a variation in the quantity of the sequence data analyzed and the factors like repeat length, the EST sequence redundancy and database mining tools, as well as the criteria used for SSRs mining [36]. In the case of some insect species, particularly lepidopterans, the development of usable SSRs is complicated because of the frequent crossing over between non-homologous SSRs leading to the exchange of flanking regions, paucity of SSRs, and history of duplication and/or multiplication events within the genome [14].

In general, EST-derived SSRs show an abundance of trinucleotide repeat motifs due to the deletions and/or additions within translated regions. There is no disturbance to the open reading frames in such cases [37]. A predominance of trinucleotide repeats has also been reported from transcriptome sequences of insects, such as *D. antiqua* (82.7%) [33], *P. solenopsis* (43.68%) [35], and *S. incertulas* (59.5%) [32]. Further, the microsatellite analysis has revealed two-fold more trinucleotide repeats (66.2%) than dinucleotide repeats (29.2%) in the genus *Neotrogla* of order Psocoptera [38]. EST-SSRs motif distribution in *M. vitrata* is also in agreement with earlier reports, including that of *Aphis glycines* [39], *P. solenopsis* [40], and *N. lugens* [41] with 29.5%, 23.38%, and 15.1% of predominant AAT/ATT repeats, respectively. On the contrary, in some insect species, such as *Venturia canescens* (62%) and *D. virgifera* (60%), a much higher percentage of AAT repeats have been recorded [42]. Interestingly, we found 26.39% of EST-SSRs with ACG/CGT repeat. The ACG/CGT motif is a highly abundant type of trinucleotide repeat in fungi and plants [16,43,44,45]. This repeat, though less abundant in insects, has also been reported in *Pachypeltis micranthus* (52 SSRs, 2.91%) [46], *Carposina sasakii* (619 SSRs, 0.65%) [47], *Varroa destructor* (2.59%) [48], *Rhopalosiphum padi* (447 SSRs, 6.01%) [49], and *Aphis glycines* (7.1%) [39] from varying orders. Tri-nucleotide motifs with an AT-rich sequence (~70%) were in higher frequency than GC-rich trinucleotide motifs in *M. vitrata* EST-SSRs. SSRs with low frequency of GC-rich motif are universal in eukaryote species, including insects [48,50,51,52]. This might be due to the methylation of CpG islands from cytosine (C) to thymine (T) by deamination [53]. Moreover, SSRs with rich AT content reduce the annealing temperature leading to higher AT-rich motifs after DNA replication slippage [52,54].

The informativeness level of a molecular marker is considered high with > 0.5 *PIC* value, moderate at 0.5 < *PIC* > 0.25, and low when *PIC* < 0.25 [55]. In the present study, a total of 14 loci showed a *PIC* value of more than 0.5, and 10 loci were 0.5 < *PIC* > 0.25, whereas the mean *PIC* was 0.45, indicating a medium-to-high level of polymorphism. A total of 19 loci were found to have the negative fixation index (*F*), whereas positive *F* values were observed in 10 other loci investigated, clearly indicating a heterozygote deficiency. Seven loci deviated significantly from *HWE*. The presence of *HWE* and alleles are pervasive in SSR studies of lepidopteran species [14,47,56]. The higher frequency of a null allele in Lepidoptera is due to the higher frequency of mutation in the flanking region because of transposable elements [57,58]. Our study showed a total of eight SSRs with multilocus amplification, and this confirms the presence of microsatellite families (multilocus SSRs) having a nucleotide sequence similarity at regions that immediately flank the tandem repeat [59,60]. Lower marker yield is a common feature of lepidopteran insects, and possible causes of this include a low genomic frequency of microsatellites, the presence of unstable flanking sequences that hinder PCR amplification, and multiple copies of flanking sequences [61]. Nonetheless, the SSRs from the present study provided good resolution, as genotypic diversity was recorded medium. In general, EST-SSRs show a relatively low level of polymorphism, which may be due to the location of these SSRs in conserved and expressed sequences compared to genomic SSRs that are spread throughout the genome [60].

Based on these selected microsatellite markers, we found that there is low to moderate genetic diversity (*Na =* 2.86, *Ne = 1*.92, *I =* 0.72, *Ho =* 0.34, *He =* 0.42) among *M. vitrata* populations from diverse agroecologies of India. Little differences between *Ho* and *He* also indicated Hardy–Weinberg equilibrium (*HWE*) and the presence of a null allele effect. The lower mean *Ho* value might be due to the Wahlund effect, the presence of null alleles, and the *HWE*-deviated population. Such levels of low genetic diversity have also been documented for other lepidopteran insects like *Diatraea saccharalis*, which was found to have *Ho* ranging from 0.08 to 0.88 with a mean of 0.42, and *He* ranged from 0.12 to 0.63 with a mean of 0.49 [61], whereas, for *Carposina sasakii, Ho* and *He* ranged from 0.00 to 0.68 and 0.06 to 0.77, respectively [47]. Population genetic structure analysis revealed low genetic differentiation (average *F_ST_* = 0.046, *p* < 0.005) and lack of genetic structure in Indian *M. vitrata*, probably due to the occurrence of high gene flow between the different sampled regions [5]. The results demonstrated that gene flow between *M. vitrata* populations in India is not confined because the genetic divergences were mainly found between the individuals. The Isolation by Distance (IBD) test revealed no significant correlation between geographic and genetic distances. Thus, *M. vitrata* in India did not confirm the isolation by distance model. The STRUCTURE analysis grouped the studied populations into two clusters (*K =* 2); however, the clusters identified no genetic structuring among the populations because no individual was strongly assigned (Q > 0.8) to be inferred as a population [8]. The PCoA and POPTREE analyses also divided all populations into two major clusters of three sub-clusters. These results suggest the communication of genetic information between different populations of Indian *M. vitrata*. A lack of genetic structure due to high migration rate has been demonstrated in several other insect species, including *Plutella xylostella* from China [8,62], Korea [63], USA [64,65], and Oceania (Australia and New Zealand) [66]; *Mythimna separata* from China [67]; *Chrysodeixis includes* from Brazil [56]; and *Argyresthia conjugella* from the Scandinavian Peninsula [68].

The lack of genetic differentiation and structure of the *M. vitrata* collected across large areas could be explained in two ways: high migratory behavior and recent population expansion. Studies on the species’ ecology and migratory behavior in India suggest that *M*. *vitrata* possesses strong flight and dispersal abilities, allowing them to travel long distances. Population dynamics studies have revealed that *M. vitrata* has strong flight and dispersal abilities, allowing them to travel long distances. Different regions show varying peak periods of *Maruca* larval populations. In North India Kanpur and Hisar, peak activity occurred from October to November, whereas in southern parts of India, peaks were observed in December and extended into January [3,69,70,71,72]. Thus, a gradual shift from North to South India was observed in the *M. vitrata* population from September to December, i.e., *Maruca* migrates North to South as winter progresses. This may be due to certain abiotic factors (weather parameters like temperature, day lengths, wind flow, etc.) influencing its adaptation and colonization at various locations in India. Thus, the natural dispersal, long-distance migration, and successive events of (re)colonization on different host crops may be the key factors contributing to increased gene flow [1,2]. The estimated gene flow values between populations also increased from North to South. This is in accordance with previous population dynamic studies discussed above. Thus, it can be inferred that *M. vitrata* undertakes long-distance migration between northern and southern India as winter progresses. This is the cause of higher gene flow (*Nm* > 1) among populations, thereby decreasing the degree of genetic differentiation and weakening the possibility of genetic drift among populations.

## 5. Conclusions

This study reports the de novo identification and characterization of 234 novel EST-SSRs, and the analysis of 25 microsatellite markers, on a set of 20 *M. vitrata* populations collected from different parts of India. The 18 polymorphic markers identified here significantly enrich the number of SSRs currently available in *M. vitrata*. These newly identified informative SSR markers can serve as effective molecular tools for population genetics studies of *M. vitrata,* including studies on gene flow, demography, biotype differentiation, and host dynamics. Moderate genetic diversity, low genetic differentiation, and high gene flow were found among the studied populations using these markers. Further, the population structure analysis showed a lack of genetic structure in Indian *M. vitrata*. This preliminary analysis supports the validation of SSR markers that will be helpful in population-level studies of *M. vitrata*. The data generated from this study will act as a valuable genomic resource in the population and migration studies of this important insect species. This will be very helpful in developing and deploying effective management strategies for this insect species and increasing food security worldwide. However, a more significant number of samplings covering its entire distribution in India and all generations occurring across the year are required to confirm further the population genetic structure and migratory behavior of this pest species in the country. The increasing knowledge of the population dynamics of *M. vitrata* will further help in pest prediction and advanced control of regional outbreaks in India.

## Figures and Tables

**Table 1:** Sampling details of *M. vitrata* populations were collected from different locations across India.

Zones	Location	Code	State	Latitude	Longitude
North East Plain Zone (NEPZ)	Dimapur	DMV	Nagaland	25.0454° N	93.0330° E
	Agartala	AGTL	Tripura	23.9137° N	91.3203° E
	Varanasi	BSB	Uttar Pradesh	25.2677° N	82.9913° E
	Kalyani	KYI	West Bengal	22.9452° N	88.5336° E
North West Plain Zone (NWPZ)	Kanpur	CNB	Uttar Pradesh	26.4400° N	80.3300° E
	Ludhiana	LDH	Punjab	30.9010° N	75.8071° E
	Hisar	HSR	Haryana	29.1416° N	75.7112° E
	New Delhi	NDLS	Delhi	28.6377° N	77.1571° E
	Pantnagar	PBW	Uttarakhand	29.0222° N	79.4908° E
Central Zone (CZ)	Jabalpur	JBP	Madhya Pradesh	23.2072° N	79.9540° E
	Raipur	R	Chhattisgarh	21.2382° N	81.7048° E
	Dapoli	DPLI	Maharashtra	17.7496° N	73.1785° E
	Latur	LUR	Maharashtra	18.4186° N	76.6161° E
	Dantiwada	DWZ	Gujarat	24.3217° N	72.3177° E
South Zone (SZ)	Bhubaneswar	BBS	Orissa	20.2650° N	85.8115° E
	Hyderabad	HYB	Telangana	17.3148° N	78.1612° E
	Guntur	GNT	Andhra Pradesh	16.3611° N	80.4348° E
	Raichur	RC	Karnataka	16.2043° N	77.3345° E
	Dharwad	DWR	Karnataka	15.4889° N	74.9813° E
	Kalaburagi	KLBG	Karnataka	17.3204° N	76.8397° E

**Table 2:** List of 25 novel SSR markers used for genetic diversity analysis in *M*. *vitrata*.

Primer ID	Forward (5′−3′)	Length (bp)	Tm (°C)	Reverse (5′−3′)	Length (bp)	Tm (°C)	Expected Product Size (bp)	Motif
MvR1	GGACGAAAAGGATGTGGAGA	20	60.05	GATGCCTCGCTGCTAACACT	20	60.57	173	(CG)6
MvR2	TGGCAGTCTCAGAAGCAGTG	20	60.33	GATGTCGGACTGGTTGTTCC	20	60.37	177	(CG)6
MvR3	ATGGGCCACACGACATAAAA	20	61.15	GCCTTGGCAGTCTCAGAAAT	20	59.43	151	(AC)6
MvR4	GGACGCACACAGACAAACAC	20	60.21	GCTCAAAGATTGCCGGTCTA	20	60.35	188	(AC)6
MvR5	GTGCCTTGGCAGTCTCAGTT	20	60.45	TAGGAACCCCTTCACAATGG	20	59.78	178	(CTT)5
MvR6	AAACTCAACAAAATGCTACCAAA	23	57.94	CAGCAGTGGAACGGAAATG	19	60.25	199	(ATC)8
MvR7	CTTGGCAGTCTCAGAGCACA	20	60.33	TTGACGTCGTAGGGGATGAC	20	60.92	186	(ATC)7
MvR8	GTAGTCGAACATCCCGCACT	20	60.14	CGCGTCATCAGGCATAGTAA	20	59.86	171	(TGA)5
MvR9	TGGCGACTCTATTGCCTTCT	20	59.98	GTTGGCTGACACATCATTGC	20	60.13	181	(GAG)6
MvR10	GGTGTGTGAAGCCATGTCAG	20	60.16	GGCCCCTTAGGCAAAGTAAC	20	59.97	172	(AAG)5
MvR11	TTGTGTGGTGACTGCGAAAT	20	60.16	CGTGTAATTTGCGTTCGTGA	20	60.70	176	(GAT)6
MvR12	CCGACTTTCACACAAAAGCA	20	59.88	CGCCCTAGTTTAGGGTAGGC	20	60.11	176	(ATC)8
MvR13	ACCCACGACTCTTGGCATAA	20	60.52	ACCAACCTGCACTTTTCCAG	20	60.15	177	(ATC)8
MvR14	CCACCATTTCCGTTGTTGTC	20	61.20	TATCCCCGGAATGTTGATTG	20	60.52	173	(TTG)5
MvR15	CCACCATTTCCGTTGTTCTC	20	60.35	GACTATCCCCGGAATGTTGA	20	59.75	176	(TTG)5
MvR16	TGACTGGCTGGAGTCATCTG	20	59.98	CTCCGATGGCAACTCATCTT	20	60.22	175	(CGA)6
MvR17	CGATGATGATGACGAAGACG	20	60.22	ACGTCTTTTTGCGATGGTTT	20	59.61	176	(GAT)8
MvR18	TGCAGCTGTTCTGGTATTGG	20	59.86	CAGCGGTCCGACTATTGTTT	20	60.13	191	(AAG)5
MvR19	GACGAATATCAAGGCGAACG	20	60.61	CGATGACAATCCCAGCACTT	20	61.07	163	(AATA)6
MvR20	GACAAGAGTGGCCATTACGG	20	60.52	TTCCGTGTCTGGGTGTGTTA	20	60.00	181	(TAAA)5
MvR21	GGCAGTCGGTTAGAAGTAGCC	21	60.28	GAGGGAAAACATGAGCTGGA	20	60.20	172	(ACAG)5
MvR22	CAGATTGCGTCCACTTTTCA	20	59.84	GAACTGTGGACCGCTGAGAG	20	61.01	175	(ATAC)5
MvR23	GTAACAGCCGTTCCGACAAC	20	60.56	TCAGGACTCCAGGTCTCACC	20	60.24	192	(AC)8(CA)9ctgacacatacacac tcacacacactgacacact(CA)7
MvR24	CCTCGCTACAAGCTCACCAT	20	60.42	ATCTGCGCGTATGTGTGTGT	20	60.22	164	(CA)6cg(CA)20cg(CA)5
MvR25	CCTTCGATGAGTCCCTGAGT	20	59.25	TGTGTGTGTGTGTGTGTGTGA	21	59.00	166	(AC)8tcacacactcact(CA)19 ctcact(CA)18cttt(CA)6

**Table 3:** Statistics of simple sequence repeats (SSRs) identified in *M. vitrata* genome.

Features	Values
Total number of sequences examined	10,053
Total size of examined sequences (bp)	5,328,011
Total number of identified SSRs	234 (2.33%)
Number of SSR-containing sequences	196 (1.95%)
Number of sequences containing more than one SSR	24 (10.26%)
Number of SSRs present in compound formation	13 (5.56%)

**Table 4:** Distribution and frequencies of simple sequence repeat types with repeat numbers in *M. vitrata* genome.

Motif Length	Repeats Number														
	5	6	7	8	9	10	11	12	15	18	19	20	23	Total	%
Dinucleotide	0	24	2	5	5	6	1	3	1	3	2	1	2	55	23.50
Trinucleotide	74	52	9	8	0	1	0	0	0	0	0	0	0	144	61.53
Tetranucleotide	17	17	0	0	0	1	0	0	0	0	0	0	0	35	14.95
Total	91	93	11	13	5	8	1	3	1	3	2	1	2	234	-
Frequency (%)	38.89	39.74	4.70	5.56	2.14	3.42	0.42	1.28	0.42	1.28	0.85	0.43	0.85	-	-

**Table 5:** Distributions of microsatellite repeat motifs in *M. vitrata* genome.

Repeat Motif	Repeats Number								
	5	6	7	8	9	10	>11	Total	Frequency (%)
Dinucleotide									
AC/GT	0	8	2	5	2	1	13	31	56.36
AT/AT	0	12	0	0	3	5	0	20	36.36
CG/CG	0	4	0	0	0	0	0	4	7.27
Total	0	24 (43.6%)	2 (3.6%)	5 (9.1%)	5 (9.1%)	6 (10.9%)	13 (23.6%)	55	-
Trinucleotide									
AAC/GTT	6	1	0	0	0	1	0	8	5.56
AAG/CTT	15	1	2	0	0	0	0	18	12.50
AAT/ATT	30	4	4	0	0	0	0	38	26.39
ACG/CGT	0	38	0	0	0	0	0	38	26.39
ACT/AGT	2	0	0	0	0	0	0	2	1.39
AGC/CTG	7	0	0	0	0	0	0	7	4.86
AGG/CCT	1	2	0	0	0	0	0	3	2.08
ATC/ATG	12	6	3	8	0	0	0	29	20.14
CCG/CGG	1	0	0	0	0	0	0	1	0.69
Total	74 (51.4%)	52 (36.1%)	9 (6.3%)	8 (5.6%)	0	1 (0.7%)	0	144	-
Tetranucleotide									
AAAG/CTTT	3	0	0	0	0	0	0	3	8.57
AAAT/ATTT	6	16	0	0	0	1	0	23	65.71
AATG/ATTC	1	0	0	0	0	0	0	1	2.86
ACAG/CTGT	4	0	0	0	0	0	0	4	11.43
ACAT/ATGT	3	0	0	0	0	0	0	3	8.57
ACTC/AGTG	0	1	0	0	0	0	0	1	2.86
Total	17 (48.6%)	17 (48.6%)	0	0	0	1 (2.9%)	0	35	

**Table 6:** Characteristics of newly identified polymorphic microsatellite markers used for genetic diversity analysis of *M. vitrata*.

Marker/ Locus	*Na*	*Ne*	*I*	*Ho*	*He*	*uHe*	*F*	*PIC*	*MAF*	*H*	*HWE*	*Null Allele*
MvR1	2	1.53	0.53	0.22	0.35	0.37	0.36	0.51	0.53	0.59	0.28	no
MvR2	2	1.96	0.68	0.00	0.49	0.51	1.00	0.59	0.40	0.66	0.21	yes
MvR3_locus1	3	1.16	0.31	0.10	0.14	0.14	−0.29	0.14	0.93	0.14	0.33	no
MvR3_locus2	3	1.53	0.63	0.24	0.34	0.35	0.32	0.47	0.68	0.50	0.24	no
MvR4_locus1	2	1.98	0.69	0.50	0.50	0.51	−0.01	0.37	0.55	0.50	0.96	no
MvR4_locus2	4	2.77	1.14	0.65	0.64	0.66	−0.02	0.57	0.45	0.64	0.57	no
MvR6_locus1	2	1.23	0.34	0.21	0.19	0.19	−0.12	0.25	0.85	0.27	0.61	no
MvR6_locus2	5	2.12	1.06	0.68	0.53	0.54	−0.29	0.54	0.63	0.57	0.88	no
MvR6_locus3	2	1.43	0.48	0.37	0.30	0.31	−0.23	0.33	0.78	0.37	0.32	no
MvR7	3	1.49	0.58	0.40	0.33	0.34	−0.22	0.29	0.80	0.33	0.74	no
MvR8_locus1	3	2.11	0.90	0.61	0.53	0.54	−0.16	0.56	0.58	0.61	0.34	no
MvR8_locus2	2	1.97	0.69	0.29	0.49	0.51	−0.04	0.53	0.48	0.61	0.10	no
MvR9	2	1.41	0.46	0.35	0.29	0.30	−0.21	0.25	0.83	0.29	0.34	no
MvR10	3	1.63	0.70	0.12	0.39	0.40	−0.07	0.50	0.65	0.53	0.18	no
MvR11	2	1.10	0.20	0.10	0.10	0.10	−0.05	0.09	0.95	0.10	0.81	no
MvR13_locus1	4	2.71	1.15	0.56	0.63	0.65	0.12	0.18	0.90	0.19	0.15	no
MvR13_locus2	4	3.27	1.25	0.57	0.69	0.72	−0.18	0.65	0.48	0.69	0.00 ***	yes
MvR14	2	1.92	0.67	0.80	0.48	0.49	−0.67	0.72	0.30	0.76	0.00 **	yes
MvR15	3	1.11	0.23	0.10	0.10	0.10	−0.04	0.36	0.60	0.48	1.00	no
MvR17	4	3.02	1.22	0.27	0.67	0.69	0.60	0.09	0.95	0.10	0.55	no
MvR18_locus1	3	2.80	1.06	0.17	0.64	0.67	0.74	0.71	0.35	0.75	0.00 **	yes
MvR18_locus2	2	2.00	0.69	0.25	0.50	0.53	0.50	0.37	0.55	0.50	0.16	no
MvR19_locus1	2	1.15	0.26	0.00	0.13	0.14	1.00	0.66	0.40	0.71	0.00 ***	yes
MvR19_locus2	5	2.75	1.22	0.78	0.64	0.65	−0.22	0.50	0.60	0.56	0.00 **	no
MvR19_locus3	3	2.11	0.90	0.39	0.53	0.54	−0.26	0.41	0.65	0.49	0.08	no
MvR24	3	1.38	0.54	0.13	0.28	0.28	0.55	0.63	0.50	0.68	0.01 **	yes
MvR25_locus1	3	2.55	1.01	0.29	0.61	0.63	0.52	0.56	0.58	0.61	0.03 *	yes
MvR25_locus2	2	1.46	0.49	0.39	0.31	0.32	−0.24	0.45	0.68	0.50	0.31	no
Mean	2.86	1.92	0.72	0.34	0.42	0.43	0.18	0.45	0.63	0.49	-	

*Na* = number of different alleles, *Ne* = number of effective alleles, *I* = Shannon information index, *Ho* = observed heterozygosity, *He* = expected heterozygosity, *uHe* = unbiased expected heterozygosity, *F* = fixation index, *MAF* = major allele frequency, *H* = gene diversity, *PIC* = polymorphism information content, *HWE p*-value for deviation from Hardy–Weinberg equilibrium (significant, * *p* < 0.05, ** *p* < 0.01, *** *p* < 0.001).

**Table 7:** Genetic diversity estimates for different geographic populations of *M. vitrata* across India.

Zones	*N*	*Na*	*Ne*	*I*	*Ho*	*He*	*uHe*	*F_IS_*	*PAL*	*% P*
NEPZ	16	1.90	1.60	0.47	0.29	0.31	0.36	0.09	2	72.41
NWPZ	20	2.17	1.72	0.55	0.32	0.34	0.38	0.02	4	79.31
CZ	20	2.21	1.80	0.58	0.34	0.36	0.41	0.04	4	75.86
SZ	24	2.28	1.76	0.60	0.35	0.37	0.43	0.08	3	86.21
Mean	20	2.14	1.72	0.55	0.32	0.34	0.40	0.06	-	68.97

*N =* number of individuals, *Na =* number of different alleles, *Ne =* number of effective alleles, *I =* Shannon information index, *Ho =* observed heterozygosity, *He =* expected heterozygosity, *uHe =* unbiased expected heterozygosity, *F_IS_ =* inbreeding coefficient, *PAL =* private alleles, *% P =* percent polymorphism, NEPZ *=* North East Plain Zone, NWPZ *=* North West Plain Zone, CZ *=* Central Zone, SZ *=* South Zone. Analyses were performed at a 95% confidence interval using GenAlEx version 6.5 software.

**Table 8:** Genetic differentiation coefficient (below diagonal) and gene flow (above diagonal) between different geographic populations of *M. vitrata* across India.

Zones	NEPZ	NWPZ	CZ	SZ
NEPZ	-	3.681	3.576	4.489
NWPZ	0.064	-	4.859	8.505
CZ	0.065	0.049	-	7.932
SZ	0.053	0.029	0.031	-

**Table 9:** Analysis of molecular variance (AMOVA) among and within different geographic populations of *M. vitrata*.

Source	*df*	Sum of Squares	Variance of Components	Percentage Variation (%)	*F* Statistics
Among populations	3	41.558	0.353	5	*F_IS_* = 0.415 (*p* < 0.001)
Among locations within populations	16	165.492	3.034	40	*F_ST_* = 0.046 (*p* < 0.005)
Within locations	20	85.500	4.275	56	*F_IT_* = 0.442 (*p* < 0.001)
Total	39	292.550	7.662	100	-

*df*: degrees of freedom, *F_IS_*: inbreeding coefficient within-population; *F_ST_*: inter-population genetic fraction coefficient; *F_IT_*: inbreeding coefficient inter-population.

## Data Availability

The data presented in this study are available in this article and supplementary material here.

## Supplementary Materials

The following supporting information can be downloaded at: https://www.mdpi.com/article/10.3390/genes14071433/s1, Table S1: Details of 79 SSR primers developed from publicly available databases of *M*. *vitrata*. Figure S1. PAGE analysis of PCR amplification product from different SSR primers.
