# Supplementary material for: De Novo Mining and Validating Novel Microsatellite Markers to Assess Genetic Diversity in Maruca vitrata (F.), a Legume Pod Borer
Source: Genes (Basel). 2023 Jul 12;14(7):1433. doi: 10.3390/genes14071433 (PMC10379186; doi:10.3390/genes14071433)
Supplement: Supplementary file 1 [file genes-14-01433-s001.zip › genes-2412902-supplementary.pdf]

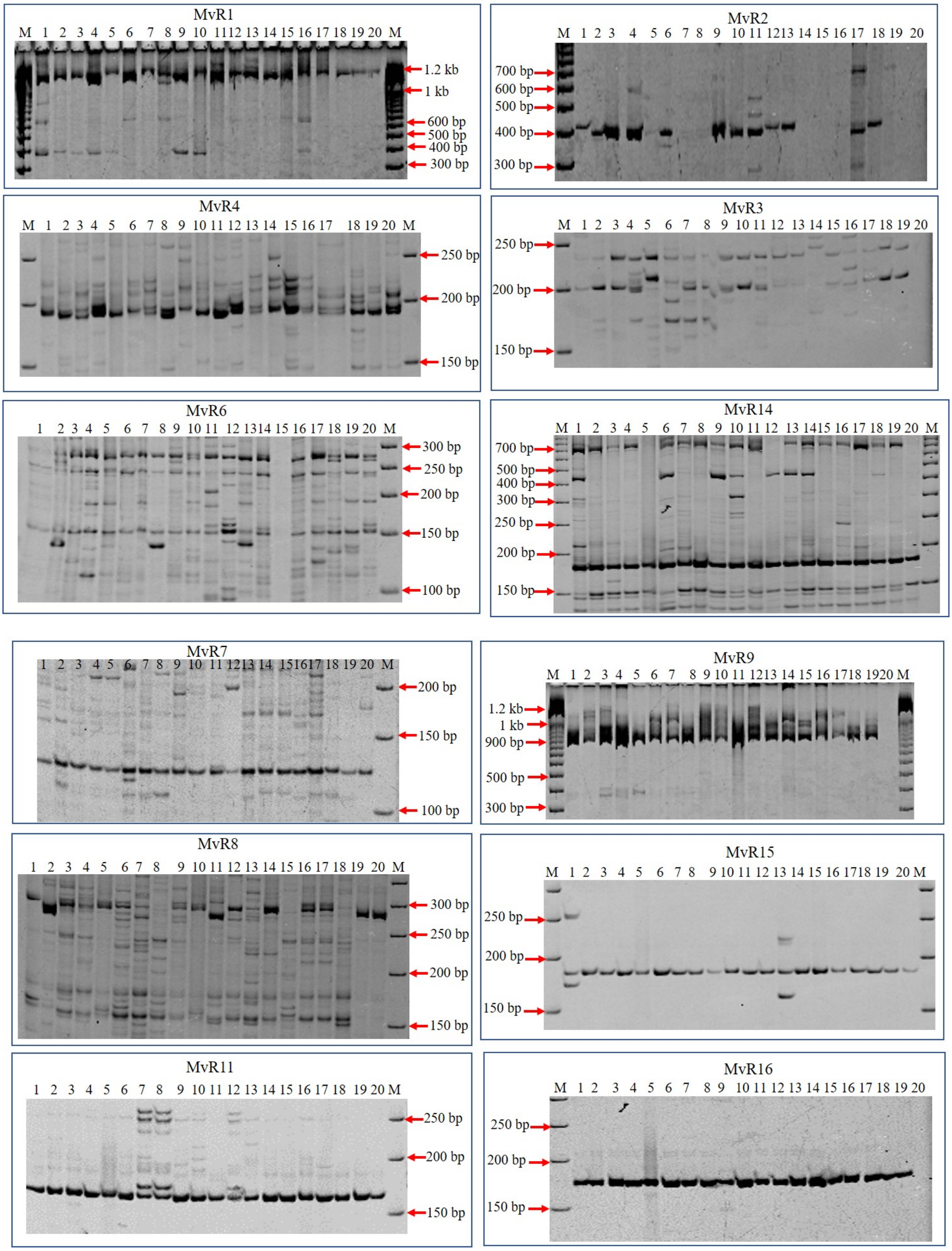
MvR1
M 1 2 3 4 5 6 7 8 9 10 11 12 13 14 15 16 17 18 19 20 M
1.2 kb
1 kb
600 bp
500 bp
400 bp
300 bp
MvR2
M 1 2 3 4 5 6 7 8 9 10 11 12 13 14 15 16 17 18 19 20
700 bp
600 bp
500 bp
400 bp
300 bp
MvR4
M 1 2 3 4 5 6 7 8 9 10 11 12 13 14 15 16 17 18 19 20 M
250 bp
200 bp
150 bp
MvR3
M 1 2 3 4 5 6 7 8 9 10 11 12 13 14 15 16 17 18 19 20
250 bp
200 bp
150 bp
MvR6
1 2 3 4 5 6 7 8 9 10 11 12 13 14 15 16 17 18 19 20 M
300 bp
250 bp
200 bp
150 bp
100 bp
MvR14
M 1 2 3 4 5 6 7 8 9 10 11 12 13 14 15 16 17 18 19 20 M
700 bp
500 bp
400 bp
300 bp
250 bp
200 bp
150 bp
MvR7
1 2 3 4 5 6 7 8 9 10 11 12 13 14 15 16 17 18 19 20 M
200 bp
150 bp
100 bp
MvR9
M 1 2 3 4 5 6 7 8 9 10 11 12 13 14 15 16 17 18 19 20 M
1.2 kb
1 kb
900 bp
500 bp
300 bp
MvR8
1 2 3 4 5 6 7 8 9 10 11 12 13 14 15 16 17 18 19 20 M
300 bp
250 bp
200 bp
150 bp
MvR15
M 1 2 3 4 5 6 7 8 9 10 11 12 13 14 15 16 17 18 19 20 M
250 bp
200 bp
150 bp
MvR11
1 2 3 4 5 6 7 8 9 10 11 12 13 14 15 16 17 18 19 20 M
250 bp
200 bp
150 bp
MvR16
M 1 2 3 4 5 6 7 8 9 10 11 12 13 14 15 16 17 18 19 20
250 bp
200 bp
150 bp

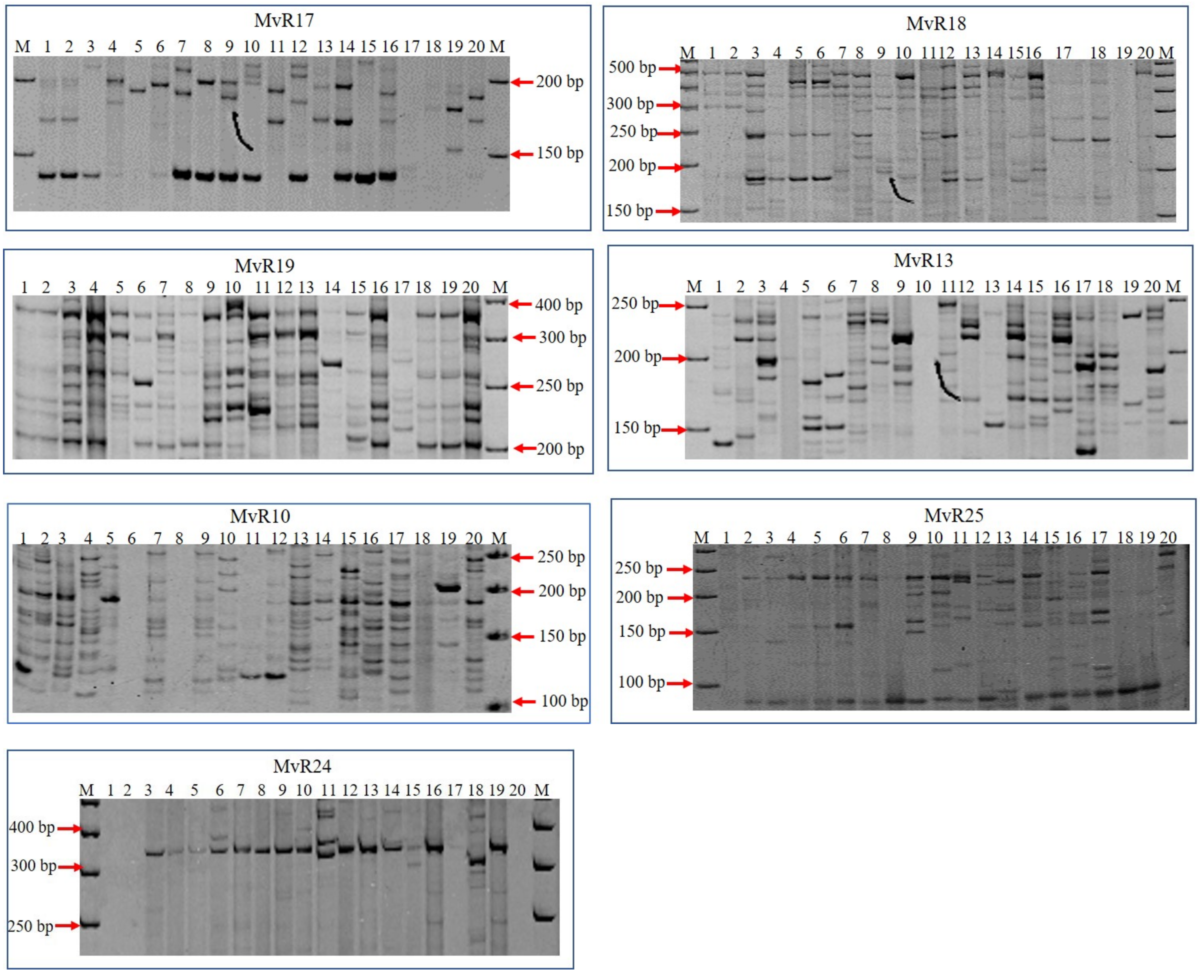

Figure S1. PAGE analysis of PCR amplification product from different SSR primers. Lane M, 50 bp DNA marker and lane 1-20 (1: DMV, 2: AGTL, 3: BSB, 4: KYI, 5: CNB, 6: LDH, 7: HSR, 8: NDLS, 9: PBW, 10: JBP, 11: R, 12: DPLI, 13: LUR, 14: DWZ, 15: BBS, 16: HYB, 17: GNT, 18: RC, 19: DWR, 20: KLBG) displayed as per Table 1 representing M. vitrata DNA samples collected from different locations from India.

Table S1. Details of 79 SSR primers developed from publicly available databases of *M. vitrata*

Index	Sequence ID	Forward	Len	tm	GC%	Reverse	Len	tm	GC%	Product size	Motif	Motif Length
1	TRINITY_DN109_c0_g1_i1	GGTGACGATGCAGAAGTTGA	20	59.84	50	GTAAGGCCCGGACCAGACT	19	61.43	63.16	170	(TGA)6	3
2	TRINITY_DN114_c0_g2_i1	ATCGCCTGGGGATAGAAGAT	20	59.89	50	ACAATGCACAAGGCAGAATG	20	59.72	45	175	(CA)8	2
3	TRINITY_DN279_c0_g1_i1	AGCGCAGTCGGTTAAAAAGT	20	59.04	45	GCGGGCCACTAATCCTTACT	20	60.47	55	172	(ACAG)5	4
4	TRINITY_DN279_c0_g2_i1	CAAATTTCATTTAAATCGGTCCA	23	60.06	30.43	GCACATGCTCAATGGTTCAA	20	60.68	45	173	(ACAG)5	4
5	TRINITY_DN206_c0_g2_i1	GTGCCTTGGCAGTCTCAGTT	20	60.45	55	TAGGAACCCCTTCACAATGG	20	59.78	50	178	(CTT)5	3
6	TRINITY_DN276_c0_g1_i1	TTTTAATCCTCCAATAGCACCAA	23	59.86	34.78	AATGGGATCAACCCCTTTTC	20	60	45	190	(ATA)5	3
7	TRINITY_DN333_c1_g1_i1	AAACTCAACAAAATGCTACCAAA	23	57.94	30.43	CAGCAGTGGAACGGAAATG	19	60.25	52.63	199	(ATC)8	3
8	TRINITY_DN333_c2_g1_i1	CTTGGCAGTCTCAGAGCACA	20	60.33	55	TTGACGTCGTAGGGGATGAC	20	60.92	55	186	(ATC)7	3
9	TRINITY_DN464_c0_g1_i1	GTAGTCGAACATCCCGCACT	20	60.14	55	CGCGTCATCAGGCATAGTAA	20	59.86	50	171	(TGA)5	3
10	TRINITY_DN469_c0_g1_i1	GATCCAAACAGGTCAACTCCA	21	59.96	47.62	TGATGGTCTGCGTAATCTGG	20	59.67	50	147	(AGC)5	3
11	TRINITY_DN471_c0_g1_i1	TGGCGACTCTATTGCCTTCT	20	59.98	50	GTTGGCTGACACATCATTGC	20	60.13	50	181	(GAG)6	3
12	TRINITY_DN471_c0_g2_i1	GTACTTCAAGAGCGGCGACT	20	59.64	55	TTGCTGTTCTCCAGCTTCCT	20	60.13	50	177	(GAG)6	3
13	TRINITY_DN466_c1_g1_i1	TCAACCAGGGAGCTTACACA	20	59.29	50	TCGGTTTAACGATGAACGTG	20	59.58	45	176	(GT)8	2
14	TRINITY_DN536_c0_g1_i1	GACGAATATCAAGGCGAACG	20	60.61	50	CGATGACAATCCCAGCACTT	20	61.07	50	163	(AATA)6	4
15	TRINITY_DN539_c0_g1_i1	GGACGAAAAGGATGTGGAGA	20	60.05	50	GATGCCTCGCTGCTAACACT	20	60.57	55	173	(CG)6	2
16	TRINITY_DN539_c0_g4_i1	TGGCAGTCTCAGAAGCAGTG	20	60.33	55	GATGTCGGACTGGTTGTTCC	20	60.37	55	177	(CG)6	2
17	TRINITY_DN549_c0_g1_i1	ATGGGCCACACGACATAAAA	20	61.15	45	GCCTTGGCAGTCTCAGAAAT	20	59.43	50	151	(AC)6	2
18	TRINITY_DN571_c0_g1_i1	AGGTGTCACAACATGCACCTA	21	59.07	47.62	CCATCTCGAGGTTAGGGTGA	20	60.07	55	188	(AAGA)5	4
19	TRINITY_DN646_c0_g3_i1	GGCCGACATTGTTTTGTTTT	20	59.85	40	TTTGTCGTCAAGATTGGAACC	21	59.96	42.86	182	(AT)10	2
20	TRINITY_DN645_c0_g3_i1	GGTGTGTGAAGCCATGTCAG	20	60.16	55	GGCCCCTTAGGCAAAGTAAC	20	59.97	55	172	(AAG)5	3
21	TRINITY_DN675_c0_g1_i1	GGCGCAAATCAGCTGTTATATT	22	60.47	40.91	GACCCCCAAAGTGGAGAAAC	20	60.73	55	182	(TCT)5	3
22	TRINITY_DN675_c0_g8_i1	GAAGGTCTGCACAACGTTCA	20	59.88	50	GCTCGTGCTGGTAAGGTCA	19	60	57.89	129	(TCT)5	3
23	TRINITY_DN668_c0_g1_i1	TTGTGTGGTGACTGCGAAAT	20	60.16	45	CGTGTAATTTGCGTTCGTGA	20	60.7	45	176	(GAT)6	3
24	TRINITY_DN668_c1_g1_i1	CCGACTTTCACACAAAAGCA	20	59.88	45	CGCCCTAGTTTAGGGTAGGC	20	60.11	60	176	(ATC)8	3
25	TRINITY_DN668_c1_g3_i1	ACCCACGACTCTTGGCATAA	20	60.52	50	ACCAACCTGCACTTTTCCAG	20	60.15	50	177	(ATC)8	3
26	TRINITY_DN695_c0_g4_i1	CCACCATTTCCGTTGTTGTC	20	61.2	50	TATCCCCGGAATGTTGATTG	20	60.52	45	173	(TTG)5	3
27	TRINITY_DN695_c0_g5_i1	CCACCATTTCCGTTGTTCTC	20	60.35	50	GACTATCCCCGGAATGTTGA	20	59.75	50	176	(TTG)5	3

28	TRINITY_DN695_c0_g8_i1	CATTGCCGTTGTTGTCACTT	20	59.62	45	CCAAACCGGTCTCTTATCCA	20	59.93	50	179	(TTG)5	3
29	TRINITY_DN716_c0_g1_i1	TCGAAAAGAAACACCGCTCT	20	59.99	45	TCTCGATCTCGGTCTGTTCA	20	59.5	50	183	(AAG)7	3
30	TRINITY_DN716_c0_g2_i1	TCCTAGGTCACGGCAAAGTC	20	60.25	55	ATTTCTTCGGAGCCTGCTTT	20	60.34	45	166	(AAG)7	3
31	TRINITY_DN742_c0_g1_i1	CAGCTGTTTCCGCAAGATTT	20	60.39	45	CAGGAGTTGGAGGAAAAGGA	20	59.25	50	193	(TG)6	2
32	TRINITY_DN742_c0_g3_i1	GGTGGCGTACGGTAGGTCT	19	60	63.16	CTCGCAAAAACAGCTGAAGA	20	59.34	45	179	(TG)6	2
33	TRINITY_DN787_c3_g6_i1	GACAAGAGTGGCCATTACGG	20	60.52	55	TTCCGTGTCTGGGTGTGTTA	20	60	50	181	(TAAA)5	4
34	TRINITY_DN755_c2_g7_i1	CGCTTCTGTGGATTCTGATG	20	59.39	50	TCAAAGTCTGTACAATCCGAAAG	23	58.44	39.13	173	(TCA)5	3
35	TRINITY_DN814_c0_g1_i1	TGACTGGCTGGAGTCATCTG	20	59.98	55	CTCCGATGGCAACTCATCTT	20	60.22	50	175	(CGA)6	3
36	TRINITY_DN814_c0_g7_i1	GCGGATGTGACGCTTCTAGT	20	60.43	55	CAGTGTGACCCAAACGAAGA	20	59.72	50	154	(CGA)6	3
37	TRINITY_DN952_c0_g1_i1	CGATGATGATGACGAAGACG	20	60.22	50	ACGTCTTTTTGCGATGGTTT	20	59.61	40	176	(GAT)8	3
38	TRINITY_DN934_c0_g1_i1	TGCAGCTGTTCTGGTATTGG	20	59.86	50	CAGCGGTCCGACTATTGTTT	20	60.13	50	191	(AAG)5	3
39	TRINITY_DN959_c0_g1_i1	GAGGACTGTCGGAATTGGAA	20	60.05	50	CTTCTTCGTCGATGTGGTCA	20	59.83	50	180	(ATG)5	3
40	TRINITY_DN959_c0_g1_i1	TGACGACGAAGACAATGCTC	20	59.99	50	TTGCTATACCACCTCATCATCA	22	58.12	40.91	141	(ATG)6	3
41	TRINITY_DN1137_c0_g2_i1	TTCCAAAACCAAAGCACCTC	20	60.09	45	AGAAAGGAACTACGCCGTCA	20	59.88	50	163	(TTG)6	3
42	TRINITY_DN1235_c0_g1_i1	CAGTGCAGGAACCATACCAT	20	58.44	50	GCGAAAACAAAACCATTGTG	20	59.06	40	141	(TTA)6	3
43	TRINITY_DN1473_c0_g1_i1	TCAGGGGGTGATTAGTGATTG	21	59.8	47.62	ATGACCTGCCCAGGAGTATG	20	59.95	55	162	(TGTC)5	4
44	TRINITY_DN1436_c0_g1_i1	CACCCGAGCAACACTATGAA	20	59.72	50	GCTGTTGTTGTTCGACAAGG	20	59.34	50	168	(GAA)5	3
45	TRINITY_DN1499_c0_g1_i1	GGCAGTCGGTTAGAAGTAGCC	21	60.28	57.14	GAGGGAAAACATGAGCTGGA	20	60.2	50	172	(ACAG)5	4
46	TRINITY_DN1883_c0_g1_i1	GCGGTGTCTATCTCGTTCCT	20	59.31	55	GGGGACAAAGGACATCCTAA	20	58.84	50	147	(GCG)5	3
47	TRINITY_DN1948_c0_g1_i1	AGTTTACGAGCGACAACTTGA	21	57.71	42.86	AGGCACGTACCTTGTTGGTC	20	60.04	55	189	(TGAA)5	4
48	TRINITY_DN1995_c0_g1_i1	TTGCAAGAGGCGTCCATAAT	20	60.61	45	GTTGGGTTTCCTGTCTTTGC	20	59.57	50	194	(AGA)5	3
49	TRINITY_DN2000_c0_g1_i1	CAGATTGCGTCCACTTTTCA	20	59.84	45	GAACTGTGGACCGCTGAGAG	20	61.01	60	175	(ATAC)5	4
50	TRINITY_DN2079_c0_g1_i1	AGGCATGATAGCGTGGAAGT	20	59.72	50	AGATGTGCGGATTACCGTTC	20	59.96	50	185	(GTA)5	3
51	TRINITY_DN2096_c0_g1_i1	TACCAGGCTGGCGTAAAACT	20	59.77	50	TCGGAACAGGTTTAAGGACAA	21	59.6	42.86	180	(AAG)5	3
52	TRINITY_DN2106_c0_g1_i1	GGCCGACATGTTTTTGTTTT	20	59.85	40	TTGCATCTCTACAGTTGATTTGG	23	59.28	39.13	200	(ATA)5	3
53	TRINITY_DN2192_c0_g1_i1	CGAAAAGCAGCTGTCAGAGA	20	59.47	50	TTTTTCTGTGGGACAAACATT	21	57.11	33.33	165	(AGA)5	3
54	TRINITY_DN2326_c0_g1_i1	GCGGCCGTACTATTGTTTTG	20	60.51	50	TGAGGATGAGGACGACAATTA	21	58.16	42.86	199	(TGTA)5	4
55	TRINITY_DN2495_c0_g1_i1	AAGGCCTATACCCTGCATTG	20	59.07	50	CAAACCCATTTAGTAAAATGTCG	23	57.72	34.78	165	(TGA)6	3
56	HS098090.1	AGCGATCGCCATTTTATAGG	20	59.19	45	ATTCCAATGCTGGCAATACC	20	59.79	45	179	(TA)6	2
57	HS097890.1	CTAGCGATGGGTCACAAAGTC	21	59.75	52.38	GCCCTTTGCTTTTCATTGTC	20	59.69	45	168	(TGA)6	3
58	HS097859.1	ACGTGGTTGCTACTGAATGG	20	58.65	50	TTCCTGAGATAAGCACGTTCAA	22	59.88	40.91	156	(CTC)5	3

59	HS097858.1	GACCATACGGACCAGGATCA	20	60.75	55	TGCTGGTCCTCCTACAATGA	20	59.24	50	182	(GCT)5	3
60	HS097699.1	TGCGAAGACGAGTGTGAGAC	20	60.19	55	TAACACCTGCTGCAAGATGG	20	59.86	50	167	(GAA)6	3
61	HS097594.1	GTCGCTGGCACAGCTAGTTT	20	60.6	55	CGAATCGCGTGTATTGTTATTC	22	59.52	40.91	176	(AATA)6	4
62	HS098802.1	TCTGGCGCTGGCTAGTTAGT	20	60.18	55	CACCTCGTAGCGTTTGAAGA	20	59.07	50	177	(AAT)5	3
63	HS098371.1	GCCCTTGCTTTGCTTTTCTA	20	59.61	45	GACACGGCTTCCCAAGATAC	20	59.56	55	182	(AAT)5	3
64	HM751150.1	AATGGGATCAACCCCTTTTC	20	60	45	TTTAATCCTCCAATAGCACCAA	22	58.63	36.36	189	(ATT)5	3
65	JN685575.1	AAAGTTGAGCAGCTTTTGTCC	21	58.64	42.86	TGCGTACCAATTCAGATTGTG	21	59.6	42.86	181	(AC)23	2
66	JN685573.1	GGACGCACACAGACAAACAC	20	60.21	55	GCTCAAAGATTGCCGGTCTA	20	60.35	50	188	(AC)6	2
67	JN685572.1	GTAACAGCCGTTCCGACAAC	20	60.56	55	TCAGGACTCCAGGTCTCACC	20	60.24	60	192	(AC)8(CA)9ctgacacatacacactcacacacactgacacact(CA)7	C
68	JN685571.1	ACACGCAGATACACCGTTCC	20	60.99	55	CTGCGTACCAATTCATGTGTG	21	60.04	47.62	158	(CA)7tacatacactcactt(AC)12	C
69	JN685570.1	TAGGGTAGGACCCAGCAGTG	20	60.13	60	ACGTTCAGCAGTGGACACAA	20	60.36	50	174	(TCT)5	3
70	JN685568.1	ACATGTGCAACGTGCATCTA	20	58.73	45	TTCCCAGACAGAATCACTGCT	21	59.86	47.62	173	(TG)11	2
71	JN685567.1	CCTCGCTACAAGCTCACCAT	20	60.42	55	ATCTGCGCGTATGTGTGTGT	20	60.22	50	164	(CA)6cg(CA)20cg(CA)15	C
73	JN685566.1	CACACACACACACACACACACA	22	61.19	50	CGATGAGTCCCTGAGTAAACAA	22	59.24	45.45	175	(AC)23atacactcactctcatacacgcaa(AC)10	C
74	JN685564.1	CACAAACAAACACAACACACACA	23	60.45	39.13	CGATGAGTCCCTGAGTAAGTG	21	57.4	52.38	184	(AC)18	2
75	JN685563.1	CCTTCGATGAGTCCCTGAGT	20	59.25	55	TGTGTGTGTGTGTGTGTGTGA	21	59	47.62	166	(AC)8tcacacactcact(CA)19ctcact(CA)18cttt(CA)6	C
77	JN685562.1	CGATGAGTCCCTGAGTAAAAATG	23	60.01	43.48	CACACACACACACAGGCAGA	20	60.43	55	180	(TG)12tatttatgtatgtatgttggatgtgagcaagtgtttgtgcatgtctgtgtctgtatctctgcc(TG)8	C
79	KP327715.1	CTTTTCATTTTTGATTCCCCAAT	23	60.38	30.43	ATCCTCCAATAGCGCCAATA	20	59.53	45	171	(ATT)5	3
